# Effect of vagal stimulation in acute asthma

**DOI:** 10.1186/2045-7022-5-S2-P13

**Published:** 2015-03-23

**Authors:** Saif Eldeen Mehmed

**Affiliations:** 1 Faculty of physical therapy cairo university pulmonary and geriatric rehabilitation Giza Egypt

## Introduction

American lung association report,there were 1.8 million emergency department visits and 3,816 deaths per year attributable to asthma [1]. according to **(GINA2012)** the role of complementary and alternative medicine in adult asthma treatment are limited because these approaches have been insufficiently researched and their effect are largely unproven [2].

the parasympathetic nervous system are the primary regulators of pulmonary smooth muscle tone. Postganglionic parasympathetic nerves mediate both cholinergic contractions and inhibitory non-adrenergic non-cholinergic (iNANC) relaxations. It is the close balance between these two that ultimately determines airway caliber. These relaxation and contraction responses are through anatomical and physiological distinct vagus nerve pathways [3].

## Aim of the work

In this presentation I want to report a new physical therapy intervention to treat acute asthma symptoms and explain the importance of team work research in asthma.

## Case description

A 67 y/o Egyptian female with a history of asthma, presents to the ER with tachypnea, and acute shortness of breath with audible wheezing. Patient has taken her prescribed medications of Cromolyn Sodium and Ventolin at home with no relief of symptoms prior to coming to the ER. A physical exam revealed the following:HR 113, RR 40 with signs of accessory muscle use. Auscultation revealed decreased breath sounds with inspiratory and expiratory wheezing and pt was coughing up small amounts of white sputum. SaO2 was 79% on room air. Visual analogue scale of dyspnea (0:10) was score of 8.

## Materials and methods

Peak flow meter,pulse oxymeter and Visual analogue scale of dyspnea (0:10) were used for pre and post treatment evaluation.treatment with vagal nerve stimulation was given with trans-cutaneous electrical nerve stimulation (TENS) device and adhesive electrodes over the carotid sheath bilateral with intensity below the muscle contraction level for 10 minutes.

## Results

Peak flows done before and after the treatment were 150/210 and auscultation revealed decreased expiratory wheezing and better airflow. 10minutes later Visual analogue scale of dyspnea (0:10) was score of zero and on auscultation there was clearing of breath sounds and much improved airflow. RR was 24 at this time, HR 83 and SaO2 was 96% on room air. Symptoms resolved and patient was sent home.

## Conclusions

Vagus nerve stimulation (VNS) can be used to reduce broncho constriction associated with asthma. Although the mechanism has not been fully established, iNANC nerves constitute a likely neural pathway since they can be activated by electrical stimulation and are known to mediate relaxation in human airways [3].

## Clinical implication

This case illustrates the potential benefit of utilizing complementary treatment as part of the management of a patient presenting with acute asthma. Utilization of this form of vagal stimulation could reduce dose of medications and its side effect. More research on this topic and other complementary therapies is recommended within a team work.

## Consent

Written informed consent was obtained from the patient for publication of this abstract and any accompanying images. A copy of the written consent is available for review by the Editor of this journal.
